# Bioelectric memory: modeling resting potential bistability in amphibian embryos and mammalian cells

**DOI:** 10.1186/s12976-015-0019-9

**Published:** 2015-10-15

**Authors:** Robert Law, Michael Levin

**Affiliations:** Department of Neuroscience, Brown University, Box G, Providence, RI 02912 USA; Department of Biology and Tufts Center for Regenerative and Developmental Biology, Tufts University, 200 Boston Avenue, Medford, MA 02155 USA

**Keywords:** Computational, Bistability, Bioelectric, Ion channels, Resting potential, Memory, Ion flux, Modeling, Xenopus

## Abstract

**Background:**

Bioelectric gradients among all cells, not just within excitable nerve and muscle, play instructive roles in developmental and regenerative pattern formation. Plasma membrane resting potential gradients regulate cell behaviors by regulating downstream transcriptional and epigenetic events. Unlike neurons, which fire rapidly and typically return to the same polarized state, developmental bioelectric signaling involves many cell types stably maintaining various levels of resting potential during morphogenetic events. It is important to begin to quantitatively model the stability of bioelectric states in cells, to understand computation and pattern maintenance during regeneration and remodeling.

**Method:**

To facilitate the analysis of endogenous bioelectric signaling and the exploitation of voltage-based cellular controls in synthetic bioengineering applications, we sought to understand the conditions under which somatic cells can stably maintain distinct resting potential values (a type of state memory). Using the Channelpedia ion channel database, we generated an array of amphibian oocyte and mammalian membrane models for voltage evolution. These models were analyzed and searched, by simulation, for a simple dynamical property, multistability, which forms a type of voltage memory.

**Results:**

We find that typical mammalian models and amphibian oocyte models exhibit bistability when expressing different ion channel subsets, with either persistent sodium or inward-rectifying potassium, respectively, playing a facilitative role in bistable memory formation. We illustrate this difference using fast sodium channel dynamics for which a comprehensive theory exists, where the same model exhibits bistability under mammalian conditions but not amphibian conditions. In amphibians, potassium channels from the Kv1.x and Kv2.x families tend to disrupt this bistable memory formation. We also identify some common principles under which physiological memory emerges, which suggest specific strategies for implementing memories in bioengineering contexts.

**Conclusion:**

Our results reveal conditions under which cells can stably maintain one of several resting voltage potential values. These models suggest testable predictions for experiments in developmental bioelectricity, and illustrate how cells can be used as versatile physiological memory elements in synthetic biology, and unconventional computation contexts.

**Electronic supplementary material:**

The online version of this article (doi:10.1186/s12976-015-0019-9) contains supplementary material, which is available to authorized users.

## Introduction

### Overview

It is well appreciated that the nervous system implements memory and information processing via electrical communication among its cells. However, bioelectric signaling is not restricted to excitable cells [1, 2]. It has long been known that all kinds of cells both generate and are sensitive to ion currents and electric fields [3–7], using some of the same ion channels and electrical synapses exploited by the CNS, but functioning on a much slower timescale. Recent data have shown that cellular resting potentials control cell behaviors such as proliferation, differentiation, and migration [8–13]. Moreover, spatio-temporal gradients of resting potential (V_mem_) are instructive, endogenous regulators of pattern formation *in vivo*, involved in oogenesis [14, 15], craniofacial patterning [16], left-right asymmetry [17–19], brain development [20], control of innervation [21], eye formation [22, 23], carcinogenesis/metastasis [24–26], regenerative polarity [27], and size control [28]. Manipulation of stable bioelectric states has enabled control of stem cell function [29–31], induction of large-scale regenerative repair [32, 33], and organ-level reprogramming *in vivo* [23].

Bioelectric gradients control morphogenetic events, regulating cell behavior via changes in downstream gene expression and chromatin state [34, 35]. This occurs via several known transducer mechanisms that convert changes in resting potential to second messenger and ultimately transcriptional responses. The voltage gradients themselves are regulated by two upstream pathways [36]. One is the variable expression of ion channels within cells. However, there is another way for gradients to be established, which does not require pre-existing transcriptional drivers.

Significant spatio-temporal changes in cell voltage distributions can occur without changes in ion channel protein or mRNA levels. This is because ion channels are gated post-translationally: existing channels can open or close due to various physiological signals, even when the transcriptome and proteome had not changed. While this is an unexpected situation in a developmental or cell biology context, it is commonplace in neuroscience, since neural networks conduct spiking dynamics purely based on the physics of ion channel activity. Action potentials do not require regulation of channel expression and networks can conduct complex electrical behavior purely at a physiological level invisible to analysis of protein or mRNA levels. Much as occurs in the brain, non-neural cells can regulate their voltage potentials by post-translational gating of channels. The gating is driven by a range of physiological events, of which perhaps the most fascinating is cell membrane potential itself. Because channels are both gated by, and determine, resting potential, this situation opens the possibility of complex regulatory feedback loops with non-obvious behavior.

Understanding such bioelectric dynamics would facilitate the construction of comprehensive models of developmental patterning [37–39], the improvement of bioelectrical interventions for regenerative medicine applications [40–42], and the design of artificial constructs for synthetic bioengineering applications [36, 43, 44]. Despite a wealth of information on the electrical properties of neurons, developmental bioelectricity is poorly understood at a quantitative level.

A key property of developmental bioelectricity is the ability of key cells to maintain specific levels of resting potential over time, changing them in response to physiological or genetic signals that trigger new phases of patterning [42, 45, 46]. Unlike most neurons, somatic cells can occupy many different stable levels of V_mem_ [47]; what features enable a given cell to maintain a specific V_mem_ range over time (stability) and to switch to a different discrete voltage level when suitably perturbed (lability)? Being able to write and re-write voltage states into cells is a key component of memory elements (e.g., flip-flops) in modern information-processing circuits, and underlies a basic mechanism of bioelectric signaling that could be widely exploited by evolution. Thus, a quantitative analysis of this kind of multi-stability is an important first step toward rational design of circuits with desired bioelectric behavior, and a key component to the formulation of quantitative, predictive models of morphogenetic events that include both physiological and transcriptional dynamics. To this end, here we present analyses designed to understand the voltage memory properties exhibited by two common model systems (mammalian cells and frog oocytes).

### Conductance models

Membrane potentials evolve in time due to currents that flow across the membrane through populations of ion channels, and these channel populations themselves activate or inactivate at rates that often depend on the membrane potential. Conductance models are systems of ordinary differential equations that allow one to simulate and interpret these interdependent processes. The elementary theory is due essentially to Hodgkin and Huxley [48], and recapitulated in some detail in Additional file 1: Section 1. The standard form for this system is in terms of current:

1 $$-C\frac{dV}{dt}={I}_{ext}+{\displaystyle \sum_i{g}_i\cdot \left(V-{E}_i\right)}$$

where *i* indexes the ion channels, *C* is the membrane capacitance, *V* the membrane potential, *I*_*ext*_ a source of external current (e.g. a voltage clamp), and *E* the reversal potential of an ion channel. Each channel conductance *g*_*i*_ takes the form

2 $$g=\overline{g}{m}^a{h}^b$$

where *a* and *b* are natural numbers and $\overline{g}$ is the maximal conductance for that channel. The functions *m*(*V*, *t*) and *h*(*V*, *t*) are themselves governed by the differential equations:

3 $${\tau}_m\frac{dm}{dt}={m}_{\infty }-m$$

4 $${\tau}_h\frac{dh}{dt}={h}_{\infty }-h$$

and the functions *x*_∞_(*V*) and *τ*_*x*_(*V*) are experimentally determined (typically by voltage clamping; [49]). The steady-state activity *x*_∞_ is typically fit to a sigmoid function ranging the interval [0,1], and the relaxation time *τ*_*x*_ is usually approximately Gaussian (e.g. [50]). Together, these two functions describe the voltage-dependence of channel kinetics.

### Definition of bistability

A fixed point in voltage is a point at which $\frac{dV}{dt}=0$, and such a point is called asymptotically stable (or an *attractor*) if, given a nearby initial voltage and time derivative thereof, the system always evolves toward that voltage value. When assessing stability by simulation, we will adopt a looser convention, saying a conductance model is bistable, or has two *memories*, if for all chosen initial voltages in the physiological range, the dynamics evolve toward one of two final voltages. Mono- and multistability are defined similarly.

### Timescales and phase portrait analysis

Some channel models are tractable to analytic methods that can guarantee multistability. To wit, the membrane timescale is ${\tau}_{membrane}=\frac{{\overline{g}}_{leak}}{C}$, corresponding to the relaxation time of a simple RC circuit with the leak channel as resistor and membrane as capacitor; so-called *fast* channel variables with *τ*_*x*_ < < *τ*_*membrane*_ are amenable to a reduction of variables *x*(*V*, *t*) ≈ *x*_∞_(*V*) [50]. When all channel variables are fast, the system can be expressed one-dimensionally as $\frac{dV}{dt}=f(V)$, and the dynamics are qualitatively captured by the phase portrait method illustrated in Fig. 2. One may verify by visual inspection that such systems have memories: those roots of *f*(*V*) where the slope $\frac{d}{dV}f(V)$ is negative.

## Methods

### Importing channel data

The Channelpedia database [51] contains a large number of models of voltage-gated ion channels derived from measurements in a variety of cell types and animals. Such a model typically consists of (at least) the constants *a*, *b* and the functions *τ*_*x*_, *x*_∞_ as well as the temperature *T* at which the experiments to determine these were performed. We retrieved 45 of these ion channel models in ChannelML format; 17 of these channels were suitable for our purposes [52–65]; see also Additional file 1: Section 2.3). It was necessary to make several corrections to database temperature values (Additional file 1: Section 2.1). We imported the channel data using the myokit toolbox [66], which also serves as a Python interface to simulations using the CVODE library [67].

### Construction of membrane models from ion channel models

#### Temperature and reversal potential

The ion channel models were recalibrated to one of two sets of mock experimental conditions, the first of these matching an amphibian oocyte preparation at 23°C and the second matching a standard mammalian preparation at 36°C. Ion concentrations and reversal potentials are given in Tables 1 and 2, respectively. We rescaled *τ*_*x*_ for each channel to reflect the model temperature using the standard temperature coefficient *Q*_10_ = 3 (see Additional file 1: Section 2.2).

**Table 1:** Chemical gradients and reversal potentials for amphibian oocyte models

Ion	Intracellular (mM)	Extracellular (mM)	Reversal potential (mV)
Na^+^	21	10	−19
K^+^	90	0.2	−156
Cl^−^	60	10.4	45
Ca^++^	0.5	0.2	−12
Na + K (for HCN channels)			−78

**Table 2:** Chemical gradients and reversal potentials for mammalian cell models

Ion	Intracellular (mM)	Extracellular (mM)	Reversal potential (mV)
Na^+^	15	145	60
K^+^	145	5	−89
Cl^−^	10	125	−67
Na^+^ + K^+^ (for HCN channels)			−15

#### Membrane constants and channel timescales

We let the membrane capacitance *C* = 1 *μF*/*cm*^2^ and the leak conductance ${\overline{g}}_{leak}=200\mu S/c{m}^2$, corresponding to a linear membrane time constant *τ*_*membrane*_ = 5 *ms*. Timescale ranges were computed for the transition region of all channel variables in the following way: the voltages at which *x*_∞_ = 0.05 and *x*_∞_ = 0.95 were computed, and *τ*_*x*_ was then evaluated at these voltages to obtain an effective timescale range for membrane variables. We classified as fast all variables whose effective timescale range fell below the membrane time constant (Table 3) non-leak channels had maximum conductance set to ${\overline{g}}_i=2mS/c{m}^2$: ten times the leak conductance.

**Table 3:** Simulated ion channels

Channel	Remarks	Reference
Cav2.1	Fast, Persistent	[57]
Cav2.2		[55]
Cav2.3		[57]
Cav3.3		[63]
HCN1	Persistent	[58]
HCN2	Persistent	[58]
HCN3	Persistent	[58]
HCN4	Persistent	[58]
Kir2.1	Fast activation	[56]
Kv1.1		[52]
Kv1.2		[61]
Kv1.4		[62]
Kv1.6		[54]
Kv2.1		[64]
Kv2.2		[59]
Nav1.3	Fast activation	[53]
Nav1.6	Fast, Persistent	[60]

See text for definition of fast variables and persistent channels

#### Combinatorial model construction and simulation

We generated models in the form of Equations (1) through (4) for a number of combinations of ion channels. We adopt a summative notation to refer to models and model classes: for instance, one arbitrary channel coexpressed with a leak channel would be *X + leak* and pairs of arbitrary channels coexpressed with a leak channel and an inward rectifying potassium channel would be *X + Y + Kir2.1 + leak*. For each channel combination, we first simulated one continuous 30-s period divided into one–second epochs. Each epoch consisted of a 50 *ms* strongly-clamped (modeled at 1000 *mS*/*cm*^2^) voltage followed by a relaxation period of 950 *ms*. Voltages were clamped in the interval [−140 *mV*, 150 *mV*], descending with epoch, with a 10 *mV* difference between consecutive epochs. Channel combinations forming finite-state memories were then tabulated based on visual inspection of the simulation results.

#### Comparison of Xenopus oocyte and mammalian cell models

We examined two basic model systems: mammalian cells and Xenopus oocytes, which are distinct due to quite different concentrations of ions inside the cells and in their respective culture media. The mammalian case will be most useful for human tissue bioengineering applications, as well as studies of channelopathy-induced birth defects [68–70] and regenerative medicine approaches. The Xenopus model is particularly well-suited for mechanistic investigations into biophysical controls of pattern formation [20, 21, 40, 71, 72], and frog oocytes are also an attractive substrate for unconventional computing or synthetic biology applications.

The channel models considered included four major families: HCN channels as well as voltage-gated sodium, potassium, and calcium channels. While the Xenopus embryo has abundant intracellular calcium, the internal calcium concentration in mammalian cells is typically low enough that depletion effects could introduce additional nonlinearities into the system’s behavior [73, 74]. However, calcium is not itself usually a voltage modifier because calcium channel expression is typically quite low compared to other channels. In fact, the classic squid giant axon model of Hodgkin and Huxley ignores calcium entirely and fits the data quite well. Unless calcium channels are overexpressed, changes in calcium concentration act as one of the many responses to V_mem_ state, alongside transduction mechanisms that include voltage-directed movement of serotonin and butyrate, activity of voltage-sensitive phosphatases, clustering of certain membrane proteins, etc. We therefore did not consider calcium channels in the mammalian models. HCN channels, while permeable to all cations, have conductances that were reported to vary with the type of transmitted ion [75]. Calcium conductance, in particular, was reported by these authors to be significantly lower than sodium or potassium conductance. We assume here that they conduct no calcium at all, but we note that simulations (not shown) suggested that HCN channels are more conducive to bistability with increased calcium conductance, at least in amphibian models.

## Results

### Nav1.6 + leak is bistable in mammalian models

**Fig. 1:**
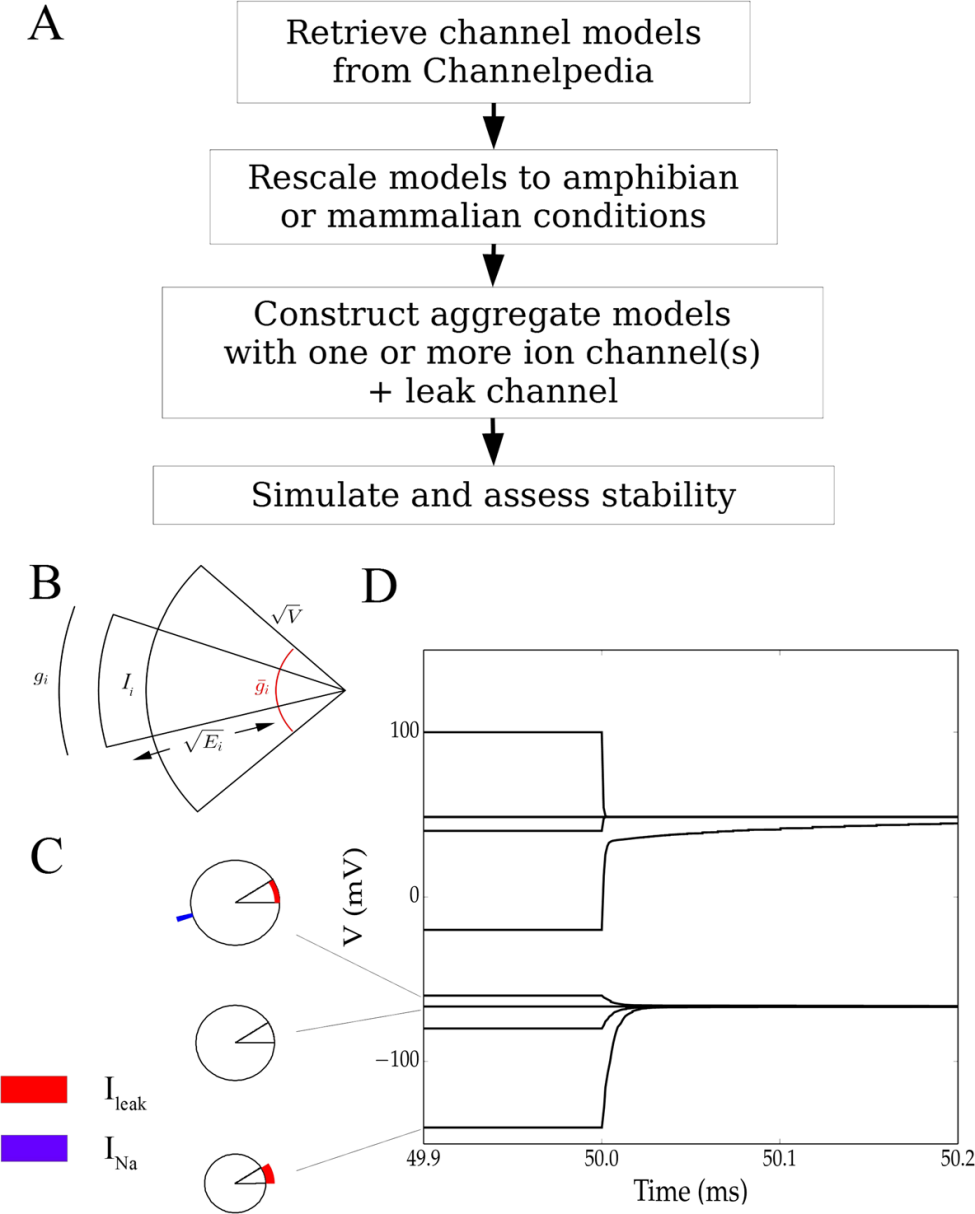
Modeling of voltage stability for ion channel combinations. Our approach for retrieving, calibrating, and simulating is diagrammed in (**a**). Currents (suppresing a factor of 2) for an arbitrary ion channel are schematized geometrically in (**b**); inward or outward currents are represented by wedge areas inside or outside the circle, respectively (**c**) Currents diagrammed using the method illustrated in **b** at three voltages above (*top*), below (*bottom*) and at (*middle*) a stable equilibrium for the Nav1.6 + leak model system, in mammals, with a 10:1 ratio of maximal conductances between Nav1.6 and leak channels. Arrows indicate the direction of voltage evolution. Note that in these diagrams, *V* and *E* have been shifted by +150 mV to be strictly positive. **d** Simulations of voltage evolution in the mammalian Nav1.6 + leak model after a 50 *ms* initial voltage-clamping period, with corresponding currents as indicated in **c**

**Fig. 2:**
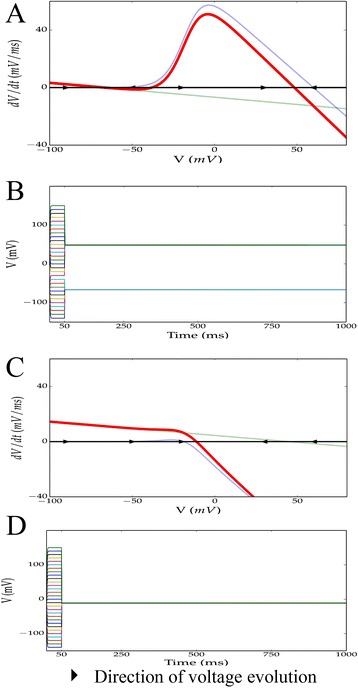
Phase-space diagrams and simulations demonstrate Nav1.6 + leak is bistable for mammalian cells but not for amphibian oocytes. We compared the phase spaces and membrane voltage dynamics for the Nav1.6 + leak model (with 10:1 maximal conductance ratio) under mammalian and amphibian oocyte conditions. **a** Phase plane for the timescale-reduced Nav1.6 + leak model for mammals and **b** corresponding simulations of voltage evolution. **c** Phase plane of the same ion channels expressed in amphibian oocyte models with corresponding simulation in (**d**). Black arrows in (**a**) and (**c**) indicate the direction of voltage evolution. Red curves indicate dV/dt as a function of V for the combined system, while blue (resp. green) indicates dV/dt supposing the Nav1.6 channel (resp. leak channel) were expressed alone. In the simulations, 30 initial voltages were chosen ranging from −140 to 150 *mV* and clamped for 50 *ms* before release. The model is bistable in the mammalian case, but monostable in the amphibian case

We first investigated in some detail the known bistability in the mammalian Nav1.6 + leak model (*cf*. the Na,p + leak model so-called in [50]), both by simulation and through the phase portrait method. After the fast channel reduction, the model is:

5 $$-C\frac{dV}{dt}=\frac{{\overline{g}}_{Na}}{1+{e}^{-0.1565\cdot \left(V+17\right)}}\cdot \left(V-{E}_{Na}\right)+{\overline{g}}_{leak}\cdot \left(V-{E}_{leak}\right)+{I}_{ext}$$

where *E*_*Na*_ = 60 *mV* and *E*_*leak*_ = − 67 *mV*. Transmembrane currents near one equilibrium are visualized directly in Fig. 1b, c and voltage evolution near clamp release is shown in Fig. 1d. The former illustrates one part of the simple underlying mechanism: at the high-voltage memory, the *Na* channels are largely open and overwhelm the leak channel current so that the voltage approaches the sodium reversal potential. At the low-voltage memory, the *Na* channels are closed, and the voltage approaches an equilibrium near the leak reversal. This system’s dynamics are further elucidated in the phase portrait in Fig. 2a, where the voltage evolves in the direction of $\frac{dV}{dt}$ (black arrowheads). Two memory voltages may be ascertained from this diagram, and simulations from 30 clamped initial conditions (Fig. 2b) confirmed that voltages evolve toward one of these two equilibria. Thus, in mammals, if Nav1.6 and leak channels are the ion channels predominantly expressed in a cell, and if the sodium channels are overexpressed relative to the leak channels, one might expect two stable memory states: one near the sodium reversal and one near the leak reversal.

### Nav1.6 + leak is monostable in amphibian oocyte models

We then examined the same model using amphibian oocyte reversal potentials *E*_*Na*_ = −19 *mV* and *E*_*leak*_ = 45 *mV*. Simulations at a number of initial conditions demonstrated the existence of only one stable fixed point, confirmed by the fact that Equation (1) has only one root with these reversal values (Fig. 2c, d). We conclude here that, unlike typical mammalian cells, amphibian oocytes will *not* exhibit bistability when Nav1.6 channels are overexpressed relative to leak channels, but rather will always evolve toward one fixed potential slightly above the sodium reversal.

### Mammalian models exhibiting bistability

**Fig. 3:**
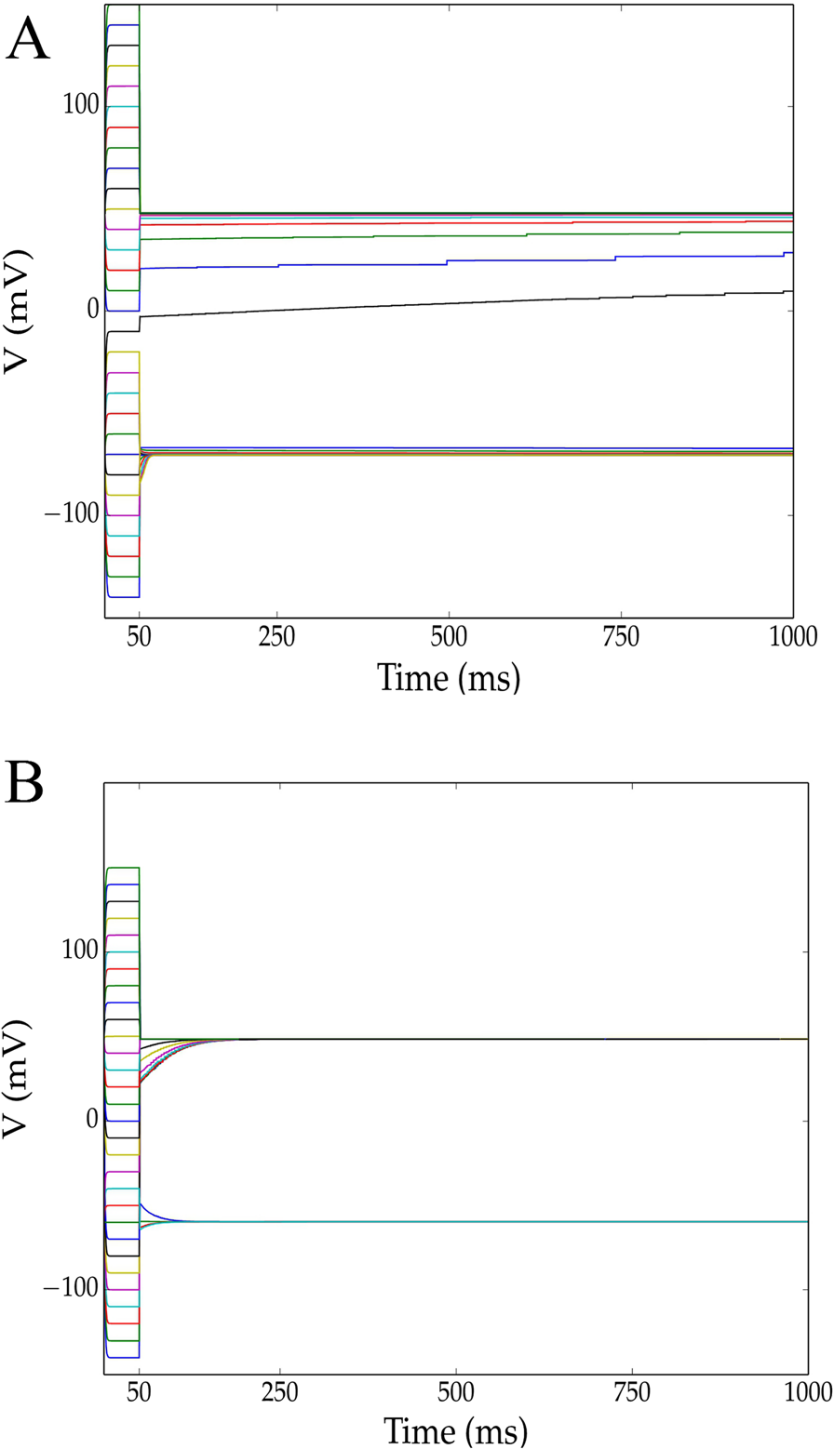
Simulations demonstrating bistability in two mammalian models. We searched for voltage bistability in X + Y + leak mammalian models (10:10:1 maximal conductance ratio) by simulation. Two such channel combinations uncovered by this search were the **a** Kv1.1 + Nav1.6 + leak and **b** HCN1 + Nav1.6 + leak models. As before, simulations were initialized at 30 voltages ranging from −140 to 150 *mV* and clamped for 50 *ms* before release. In both of these models, voltages initialized in the physiological range converge on one of two locally stable voltages

We then searched for other ion channel combinations that would lead to multistable memories under mammalian ionic conditions by simulating all X + leak and X + Y + leak models. In the X + leak scenario, the only channel combination leading to bistability was the Nav1.6 + leak model above. Bistable channel sets in X + Y + leak models are shown in Table 4. Inclusion of Nav1.6 was necessary, but not sufficient, for bistability of all of these models: introducing certain potassium channels (*n.b.* we considered only those from the Kv1.x and Kv2.x families; see Section 2.3 in the Additional file 1) tended to disrupt bistability. Combinations X + Y + Nav1.6 + leak and X + Y + Z + Nav1.6 + leak did not lead to multistability with more than two memories. Bistability was preserved after introducing more channels in many cases, for instance in Kv1.1 + Nav1.6 + leak (Fig. 3a). Note that the convergence rate is slow in this case owing to the slow dynamics of the Kv1.1 channel. These simulations suggest that bistability may be experimentally realized in mammalian cells simply by increasing the expression of Nav1.6 channels.

**Table 4:** Channel sets forming bistable memories in mammalian cell models and corresponding memory loci

X + Nav1.6 + leak	Memory loci (mV)
-	−67,48
HCN1	−60,48
HCN2	−65,48
HCN3	−60,48
HCN4	−60,48
Nav1.3	−67,48
Kv1.1	−70,48
Kv1.4	−71,48
Kv2.1	−67,48
Kv2.2	−67,48
Kir2.1	−83,48

### Amphibian models exhibiting bistability

**Fig. 4:**
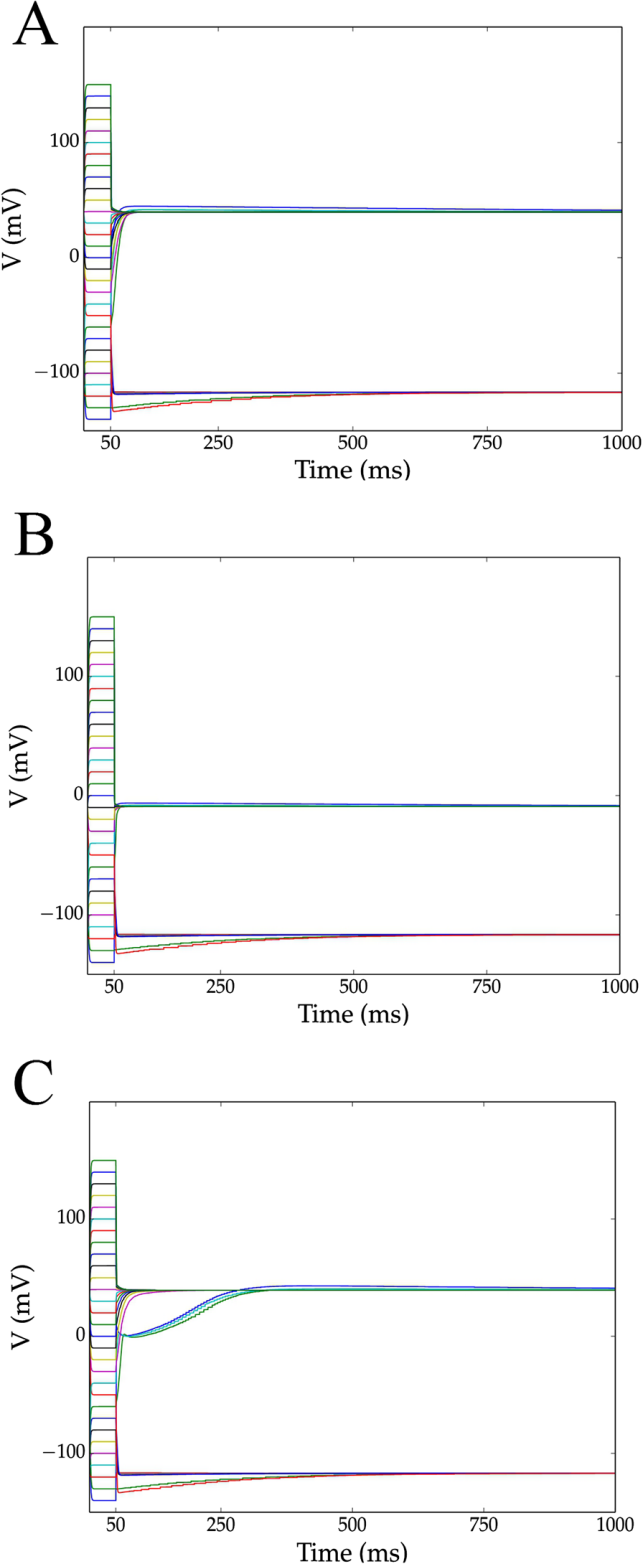
Simulations demonstrating bistability in three amphibian models. We searched for voltage bistability in X + Y + leak amphibian oocyte models (10:10:1 maximal conductance ratio) by simulation. Simulations were initialized at 30 voltages ranging from −140 to *150 mV* and clamped for *50 ms* before release. Three such channel combinations were the **a** Kir2.1 + leak, **b** Cav2.1 + Kir2.1 + leak, and **c** Cav2.2 + Kir2.1 + leak models. Note that Cav2.1 is a fast channel, and that A and C have effectively identical memory loci. In all of these models, voltages initialized in the physiological range converge on one of two locally stable voltages. The basins of attraction for these stable voltages are however, disconnected (in V), as initial hyperpolarization to –140 *mV* resulted in evolution to the higher-voltage memory

We repeated the simulations from the previous section under amphibian oocyte conditions (Table 5). Kir2.1 in the amphibian case plays a very similar role to Nav1.6 in the mammalian case. Inward rectifying potassium channels formed memories when combined with a leak channel (Kir2.1 + leak; Fig. 4a). Among the channel pairs (X + Y + leak) we considered, the presence of inward-rectifying potassium channels was necessary, but again insufficient, for bistability. Here, the presence of other potassium channels had a strong tendency to disrupt bistability. Again, attempts to construct higher-order memories using 3-tuples of these channels with Kir2.1 (up to X + Y + Z + Kir2.1 + leak) did not yield any n-state memory with n > 2. Consideration of timescales pointed to a correspondence between fast channels and memory location in the amphibian case. When memory-forming pairs included a fast channel (indicated with an superscript ‘a’ in Table 5), one memory was found near the reversal potential of that channel’s corresponding ion (e.g. the Cav2.1 + Kir2.1 + leak model in Fig. 4b).

**Table 5:** Channel sets forming bistable memories in amphibian oocyte models and corresponding memory loci

X + Kir + leak	Memory Loci (mV)
-	−117,39
Cav2.1^a^	−117,−9
Cav2.2	−117,39
Cav2.3	−117,39
Cav3.3	−117,39
HCN1	−93,39
HCN2	−96,39
HCN3	−94,39
HCN4	−96,39
Kv1.4	−117,39
Nav1.3	−117,39
Nav1.6^a^	−117,–16

^a^Indicates a fast channel

### Slow variables may lead to overshooting of nearby attractors

Importantly, the initial conditions that lead to particular equilibria do not always form connected regions in voltage space - in other words, after release, a clamped system need not evolve to the nearest voltage memory but instead may deterministically overshoot a nearby attractor due to the presence of slow variables (Figs. 3b, 4a, 6c). If these models were reproduced to reasonable accuracy, driving a system toward a low voltage (for instance by opening a strongly expressed light-sensitive potassium channel population; see, e.g. [76]) should cause the system to jump to a high-voltage memory, rather than the nearby low-voltage memory, after cessation of the driving current.

### Channel combinations not exhibiting multistability

**Fig. 5:**
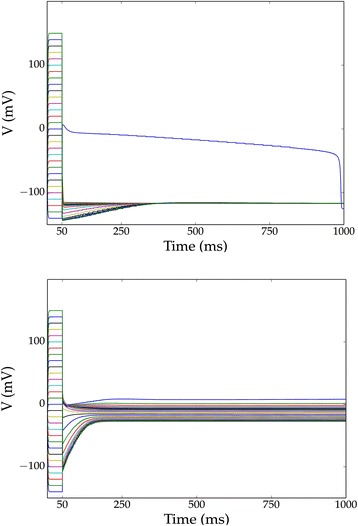
Examples of simulations not exhibiting bistability. We examined instances of models generating non-bistable behavior in the X + Y + leak amphibian case (10:10:1 maximal conductance ratio). Simulations were initialized at 30 voltages ranging from −140 to 150 *mV* and clamped for 50 *ms* before release. **a** The Kv1.2 + Kir2.1 + leak model exhibits a hyperpolarization-evoked transient caused by clamping at –140 *mV*. **b** The Cav2.2 + Kv2.2 + leak model does not appear to be asymptotically stable

For completeness, we remark on some channel combinations where multistability did not occur, or where convergence was not observed or inferable within a 10 *s* timeframe. A majority of these were monostable, e.g. the system in Fig. 5a, where slow channel activation and inactivation led to a transient depolarization but eventual convergence on equilibrium. Other models, however, did not appear to converge on any particular asymptotically stable equilibrium, a phenomenon that often occurred in the presence of Kv channels in amphibian models (see e.g. Fig. 5b). Indeed, with the exception of Kv1.4, Kv channels always disrupted bistability in amphibian models and often disrupted bistability in mammalian models as well. This points to a heuristic where suppression of Kv channels should in general aid in the experimental realization of membrane potential multistability.

### Reversible switching between memory loci

**Fig. 6:**
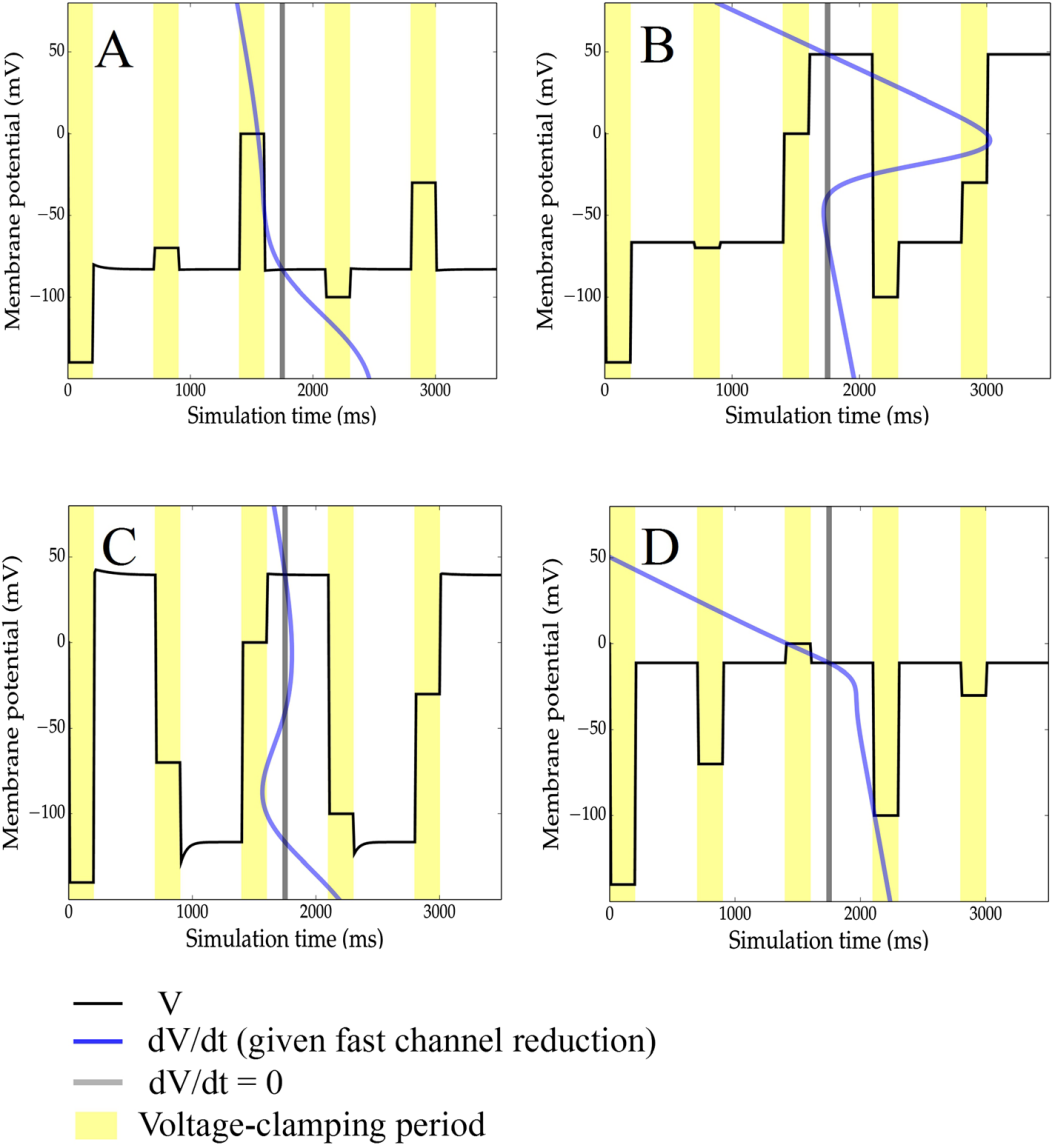
Switching between stable voltages. We simulated a sequence of voltage clamps at the following values: −140 *mV, −*70 *mV*, 0 *mV, −*100 *mV, −*30 *mV*, for several models: **a** mammalian Kir2.1 + Nav1.3 + leak, **b** mammalian Nav1.3 + Nav1.6 + leak, **c** amphibian oocyte Kir2.1 + Nav1.3 + leak, and **d** amphibian oocyte Nav1.3 + Nav1.6 + leak. Clamping periods are in yellow and phase-space diagrams are overlaid, with the grey line indicating *dV*/*dt* = 0 (see also Fig. 2). In the bistable models (**b** and **c**) the current introduced by the voltage clamp may be seen to act as a switch between stable equilibria, while in the monostable models (**a** and **d**), releasing the clamp always leads to the same equilibrium point

Finally, we illustrate controlled switching between stable memories, as might be experimentally realized by sequences of applied transient currents. Here, we simulated one continuous 3.5 s period divided into 700 *ms* epochs. In each epoch, the voltage was clamped for 200 milliseconds and released for 500 milliseconds, with the following sequence of clamped voltages chosen, in *mV*: −140, −70, 0, −100, −30. As above, the membrane potential rapidly evolves to stable fixed points after the clamp is released (Fig. 6), demonstrating that it is possible to reversibly drive the voltage from one attractor to the other, implementing a true memory element.

## Discussion

Bistability has been described previously [50, 77–82], although data have been focused on neurons. Our findings extend prior knowledge not only by examining a much larger array of ion channels, but also by comparing the contributions of these channels to bistability in both amphibian oocytes and mammalian systems beyond neurons. In so doing, we highlight a major difference in voltage dynamics between these classes arising due to differences in reversal potential loci. The work we have presented here may also be seen as a rough guide for the synthesis of cells capable of bistable voltage patterning through appropriate choices of ion channel expression.

Although our models assert a specific 2 *mS*/*cm*^2^ maximal conductance for its nonleak ion channels and a 10:1 nonleak to leak conductance ratio, our results are likely to hold approximately when either Nav1.6 or Kir2.1 channels are overexpressed relative to a cell’s other ion channels, although the values of *V*_*mem*_ may differ. We furthermore expect that small differences in reversal potentials should not make a major difference in stability properties (e.g. in Fig. 2, changing a reversal potential by a few millivolts will not affect the number of roots); the profound difference seen between amphibian oocyte and mammalian models is not due to minute differences in reversal potentials; the reversal potentials for mammals and frog oocytes are not even in the same *order*.

Interestingly, the specific channels we identified are already implicated in examples of bioelectric control of patterning. For example, Kir2.1 is known to regulate muscle cell differentiation [83–86] and craniofacial patterning (being responsible for Andersen Tawil Syndrome and its craniofacial deformities [87–89]). Likewise, the NaV1.6 channel is implicated in neural development [90–92].

The ion channels we have simulated, of course, do not represent channels in totality, and our work highlights the need for further contributions to databases such as Channelpedia; Nav1.2 and Nav1.5, for instance, are also strongly implicated in patterning [33] and cellular controls [93–95] but not found in this database. More complete repositories of channel models should facilitate further discoveries based on combinatorial approaches such as this one. However, given the set we have simulated, the relative rarity of channels capable of generating bistable voltage patterns raises the possibility that three- and higher-fold stability is *not possible* for uncoupled cells in uniform bath conditions without the aid of regulatory processes or modulators that alter the level of ion channel expression or efficacy. This hypothesis would be falsified if a combination of fast ion channels could be found such that the Equation (1) has 5 or more zero crossings with derivatives of alternating sign. If membrane voltage multistability indeed represents a switch affecting cell metabolism, then a deeper search for higher-fold stability would certainly be worthwhile. This, however, may depend on the relative expression levels of different ion channel populations. Maximizing the number of memories for an arbitrary set of fast ion channel models is equivalent to the following optimization problem: $\underset{\overline{\mathbf{g}}}{ \arg \max}\left|r\left(f\left(V,\overline{\mathbf{g}}\right)\right)\right|$, where *f* is the right hand side of equation (1), $\overline{\mathbf{g}}$ is a vector of maximal conductances, and |*r*(⋅)| counts the real roots of a function.

However, it is not necessary that individual cells shoulder all the burden for multistability. Given that evidence indicates many locally stable voltages existing concurrently in frog embryos, it is likely that gap-junction coupling plays a much larger role *in vivo* than in the relatively simple ion channel dynamics we have depicted here. Moreover, the memories we have approached here are fixed points: zero-dimensional subspaces of the phase space. There is certainly no reason that higher-dimensional attractors - stable voltage oscillations, for instance - should not also be considered as candidates for functional memories, although the means of transduction between membrane potential oscillations and cell metabolic processes is less obvious. Finally, it should be noted that *in vivo*, some cells are able to regulate both internal and external ion levels as well as the complement of ion channels, allowing them to fine-tune the stability points of any voltage memories. Future studies must examine the constraints and capabilities of such self-tuning electric networks in the context of multicellular tissues.

Our analysis has specified some conditions under which stable voltage memories can exist. We have also identified specific ion channels that can form the elements of a synthetic biology toolbox for implementing rewritable bioelectric memories. Moreover, our analyses have yielded a number of surprising findings that should be kept in mind when formulating models of developmental bioelectric patterns or exploiting voltage states in bioelectricity. Foremost is the difference found between ion channel roles in amphibian oocytes and mammalian cells, an interesting standalone result from the perspective of comparative physiology; it may also be a useful guide for future bioengineering and regenerative medicine studies that develop bioelectric strategies in Xenopus embryos and work to move them towards mammalian applications. Following this is the intriguing finding of hyperpolarization-evoked evolution to depolarized memories, which underscores the need for quantitative modeling: it cannot be assumed that induced expression of a normally hyperpolarizing channel will necessarily lead to stable hyperpolarization. A third interesting finding is the ability of Kv channels to disturb bistability, although this was not entirely consistent across the models assessed here.

Our work here was restricted to ion channels with first-order kinetic models for their activation and inactivation variables; further research might include channels like Kv1.5, which cannot be modeled effectively in this way [96]. Additional factors that influence V_mem_, to be incorporated into future models, could include additional conductances (leak channels, pumps, gap junctions, calcium stores), modulators of lipid bilayer capacitance, and physical regulators of channel activity such as pressure/tension, temperature, and chemical ligands. It is also crucial to extend this analysis to the study of multicellular bioelectric networks – cells coupled with gap junctions (electrical synapses [97–99]), which will doubtlessly have even more interesting and complex properties than single cell models. Likewise, it will be important to understand the dynamics of interacting membrane domains *within* individual cells [100, 101], which arise from diverse ion channel protein cargo on lipid rafts as well as from ion sequestration caused by complex cell geometries. Although reagents for functionally addressing such subcellular domains are currently limiting, the development of optogenetics and other high-resolution techniques will soon make it essential to test hypotheses of information coding by distinct voltage states on the surface of single cells (as occurs in the more familiar and linearly-extended neurons).

## Conclusions

In general, bioelectric state is an important instructive, tractable regulator in cancer, embryonic patterning, regeneration, and stem cell regulation [9, 12, 36, 42, 45, 102]. Our main points are the following. 1) Bistable switches may be constructed by overexpressing Kir2.1 in amphibian oocytes and Nav1.6 in mammalian cells, and by limiting expression of many of the non-inward-rectifying forms of voltage-gated potassium channels. 2) Cells with strongly expressed fast channels will tend to have memory loci near the reversal potentials for those channels. 3) Memory elements may still be constructed using slow channels, but these should not be expected to have contiguous basins of attraction. These basic principles suggest a number of predictions that will be tested in vivo in developmental bioelectricity experiments to understand endogenous patterning and also serve as guidelines for bioengineering applications. Under the right conditions, many types of somatic cells should be able to operate as physiological memory elements. Thus, the quantitative, predictive understanding of bioelectric dynamics in single cells and multicellular circuits outside of the central nervous system will be an enabling step for new pattern control applications in regenerative biomedicine and synthetic bioengineering.

## Additional file

Additional file 1: **Supplementary Materials.** (PDF 261 kb)
