# Supplementary material for: Bioelectric memory: modeling resting potential bistability in amphibian embryos and mammalian cells
Source: Theor Biol Med Model. 2015 Oct 15;12:22. doi: 10.1186/s12976-015-0019-9 (PMC4608135; doi:10.1186/s12976-015-0019-9)
Supplement: Supplementary file 1 — Supplementary Materials. (PDF 261 kb) [file 12976_2015_19_MOESM1_ESM.pdf]

Supplementary Materials

1 Theory for conductance models

We refer mainly to [10] in this section.

A patch of a cell membrane may be viewed as an electrical circuit (Supplementary Figure 1) quantifying both the ion flow across it and the accumulation of ions near the membrane surface. It must first be shown that each component of this *equivalent circuit* corresponds to a particular current, and then, using the principle of charge conservation (Kirchoff's current law), we will group all these currents into a differential equation describing the evolution of voltage over time.

A current will be defined as positive (or inward) either when positive ions flow into the cell or when negative ions flow out. Note that all variables except for voltage may be restated as densities, e.g. current per unit area, but we omit these distinctions in this section for clarity.

1.1 Ion channel currents

Each class of ion channels is treated as a battery with variable internal conductance g_i and current I_i related by $g_i \triangle V = I_i$. The conductance for the ion channel population depends on the probability of a channel in that population is open, so we have:

$$g_i = \overline{g_i} p_i$$

where $\overline{g_i}$ is the maximal conductance (when all ion channels in class i are open) and p_i is the probability that any given channel in that class is open. The *configuration* of a set of ion channels will refer to the set of these conductances. We can visualize configurations in Figure 1B-C in the main text, where we represent the proportion of open channels over total channels as angles on a circle. So-called "leak" channels are always open, so the total current is a simple linear relationship with the voltage (i.e. it follows Ohm's law). Other channel classes may open with probabilities that depend on membrane voltage (see also Section 1.1.4)[1] .

The battery represents the concentration gradient's effect on the flow of an ion (or ions, if the channel is permeable to more than one [6]), its voltage equal to the channel's reversal potential E_i. At the reversal potential, diffusion down the concentration gradient is counterbalanced by the electrical force and the current is equal to zero.

The current flowing across each population of ion channels, then, is:

$$I_i = g_i \cdot (V - E_i) \tag{1}$$

[1] Other external factors that may affect ion channel gating, e.g. pressure, may also lead to nonlinearities, but the system will usually be described as *nonautonomous*, or explicitly depending on time.

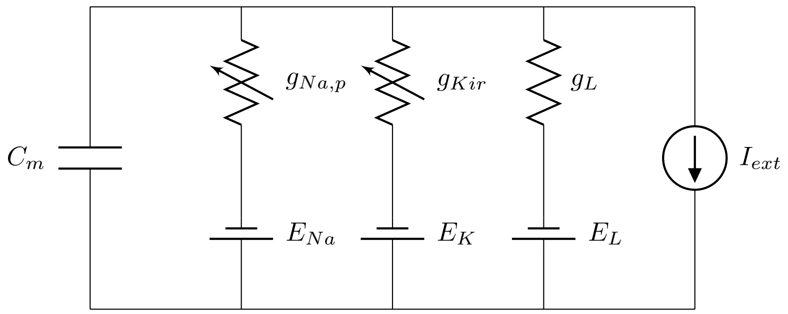

Figure 1: Equivalent circuit for a membrane patch with two voltage-gated channels, one leak channel, and external current source

1.1.1 Capacitive current

The phospholipid bilayer, meanwhile, acts as a capacitor, allowing for a charge Q to collect on either side of the membrane depending on the voltage V across it. The amount of charge it accumulates per unit voltage, which depends on its surface area, is its *capacitance* $C = \frac{Q}{V}$. Whenever the voltage across the membrane changes, this charge redistributes, generating a capacitive current I_c. Because $I = \frac{dQ}{dt}$ by definition, rearranging and differentiating with respect to time (assuming constant capacitance) yields an alternate description of this current in terms of capacitance and the rate of voltage change:

$$I_c = C\frac{dV}{dt} \tag{2}$$

1.1.2 Current balance

Kirchoff's current law is a statement of the charge conservation principle for ideal conductors (here, small stretches of intra/extracellular fluid between the membrane and measurement apparatus), represented by wires/short circuits in the circuit diagram. No current "disappears" at any point on these conductors, and so any inflowing (positive) current must be balanced by outflowing (negative) current at each point. This balance of all currents (indexed by j) I_j is captured by the equation:

$$\sum_j I_j = 0$$

which in our case, after rearranging, results in a *differential equation*:

$$-C\frac{dV}{dt} = I_{ext} + \sum g_i \cdot (V - E_i) \tag{3}$$

where I_{ext} represents current from an external source such as a voltage clamp.

1.1.3 Individual channel currents and phase portraits

As indicated in Equation 1, each channel i's current can be viewed as drawing the voltage toward E_i at a rate that increases with distance from the reversal potential; this rate is then scaled by the number of open channels. The reversal potential changes with internal and external ion concentrations, as well as temperature, both of which differ in the amphibian (oocyte) and mammalian cases.

Gating variables In order to model the dynamics of specific active channels, we next introduce *gating variables* x, usually classified into m and h, that describe the voltage's probabilistic opening and/or closing of channels through the reconfiguration of channel proteins. Each gating variable acts independently so that their probabilities multiply; a channel with $p = m^3h$ has three activation gates and one inactivation gate. Channels without inactivation gates are called *persistent.*

Activation and inactivation have different voltage dependencies and act on different timescales, and this allows a channel population to open rapidly as the voltage crosses, say, $-45mV$ and slowly close once it crosses $70mV$. A channel's activation properties can often be modeled using two experimentally determined functions: $m_\infty(V)$ and $\tau_m(V)$, with inactivation (h_∞ and τ_h) treated similarly[2]. These gating variables can be seen as opening or closing with time according to the differential equation:

$$\frac{dx}{dt} = \frac{x_\infty(V) - x(V,t)}{\tau_x(V)} \tag{4}$$

This equation models the effect of voltage clamping. When the voltage V is held fixed, the channel population eventually approaches an activation or inactivation probability x_∞, with the distance between x and x_∞ decreasing at an exponential rate $\frac{1}{\tau_x}$. Its multiplicative inverse, the relaxation time τ_x, is also known as the time constant (despite varying with V).

$m_\infty(V)$ is usually *monotonically increasing* with V, so that increasing membrane voltage tends to activate more channels. An important class of exceptions have monotonically *decreasing* $m_\infty(V)$. The $K_{ir}2.1$ channel and the HCN family are examples of these so-called *inward-rectifying* channels.

1.1.4 Fast channels

Some channels - the so-called *fast channels* - reach m_∞ essentially immediately and are known to have a role in voltage memories. A channel is considered fast if [3] τ_m is near zero, making $m(V,t) \approx m_\infty(V)$. This can be seen by rearranging Equation 4 to $\tau_m \frac{dm}{dt} = m - m_\infty$. Here, m is no longer time-dependent; in fact, it is no longer a differential equation but a *function.* For such channels, evaluating

[2] We may use the variable x to refer to either m or h.

[3] One can imagine the membrane and leak channel as a low-pass filter suppressing dynamics faster than the membrane timescale.

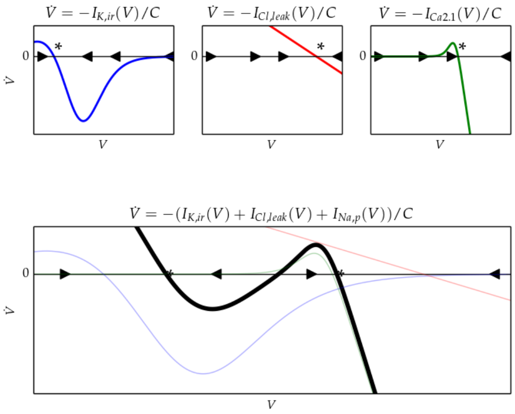

Figure 2: Top: from left to right, phase portraits dV/dt vs. V for a cell expressing only Kir2.1, leak, or Ca2.1 channels (all channel variables are assumed fast for illustrative purposes). Bottom: phase portrait for a cell expressing all these channels (10:1:10 maximal conductance ratio), with individual cell $\dot{V}$ overlaid. Black arrows indicate direction of voltage evolution. Stable fixed points are indicated by *

dV/dt is a simple matter of plugging in $m_\infty(V)$ for m where it appears in the current equation (e.g. [10, Ch. 3]).

When all channels are fast, the entire system of differential equations becomes one dimensional, expressible entirely in terms of V. Writing this as

$$\frac{dV}{dt} = f(V) \tag{5}$$

we can now see that the memories exist precisely where $f(V)$ crosses the V axis with a downward slope. At voltages slightly above V_{mem}, $\frac{dV}{dt}$ is negative, and the membrane potential returns to V_{mem}. Slightly below V_{mem}, $\frac{dV}{dt}$ is positive and If $\frac{dV}{dt}$ is negative at very high voltages and positive at very low voltages, as is always the case here, then the dynamics are guaranteed *a priori* to evolve toward precisely one memory. Moreover, the number of memories is bounded from above by $\lceil R/2 \rceil$, where R is the number of roots of $f(V)$.

When channels are not fast, it is not guaranteed that m ever reaches or even approaches m_∞ under unclamped conditions. It is not necessarily true, however, that only fast channels may form multistable memories, as we observe in the main text.

name	ion	temp	rev	m exponent	h exponent
A	UA	23	-65	3	1
Ca	Ca	35	50	2	1
Cav2.1	Ca	21	135	1	0
Cav2.2	Ca	36	135	2	1
Cav2.3	Ca	36	135	1	1
Cav3.1	Ca	0	30	1	1
Cav3.3	Ca	0	30	1	1
HCN1	Hcn	0	-45	1	0
HCN2	Hcn	0	-45	1	0
HCN3	Hcn	0	-45	1	0
HCN4	Hcn	0	-45	1	0
Ih	Hcn	34	-45	1	0
K	K	9.3	-78	4	0
K	K	23	-65	2	1
K	K	23	-65	2	1
K	K	23	-65	1	1
Kir2.1	K	28	-70.6	1	2
Kv1.1	K	24	-65	1	2
Kv1.2	K	20	-65	1	1
Kv1.3	K	0	-65	1	1
Kv1.4	K	0	-65	1	1
Kv1.5	K	9	-90	1	1
Kv1.6	K	23	-65	1	1
Kv2.1	K	23	-65	1	1
Kv2.2	K	23	-65	1	1
Kv3.1	K	23	-65	1	0
Kv3.1	K	22	-80	4	1
Kv3.1	K	23	-65	1	0
Kv3.2	K	22	-80	2	0
Kv3.3	K	23	-65	1	0
Kv3.3	K	23	82	2	1
Kv3.4	K	23	-65	1	1
Kv4.2	K	23	-68.7	3	1
L	Ca	36	80	2	1
L	Ca	36	80	2	1
M	K	0	-84	1	0
M	K	0	-84	1	0
M	K	0	-84	1	0
Na	Na	9.3	50	3	1
Na	Na	23	50	3	1
Na	Na	23	50	3	1
Na	Na	23	50	3	1
Na	Na	23	50	3	0
Nav1.3	Na	23	50	3	1
Nav1.6	Na	23	50	1	0

Table 1: Ambient temperatures and reversal potentials for ion channels as listed in the Channelpedia database. HCN: Hyperpolarization-activated and cyclic nucleotide-gated (responsive to both voltage and internal cAMP, where cAMP shifts the voltage response)

2 Model selection, parameter correction, and standardization

45 ion channel conductance models were found as .channelml files in the Channelpedia [15] database and imported into the `myokit` toolbox [4]. Some basic model information - as found in the database - is shown in Table 1. These models had been constructed from a number of different voltage-clamp experiments conducted at several different ambient temperatures. Some entries in this database were incorrect, and our goal in this section is first to make necessary corrections, and then to standardize these models to a particular temperature so that channel models generated under different conditions may be combined.

2.1 Temperature Corrections

Several channels were indexed in the database with an implausible experimental temperature of $0°C$ - examination of a source publication suggested that this

Channel name	Corrected T(C)	Mammalian	Original reference	Temperature reference
Cav3.1	25	N	[20]	[1]
Cav3.3	25	N	[20]	[1]
HCN1	36	Y		
HCN2	36	Y		
HCN3	36	Y		
HCN4	36	Y		
Kv1.3	36	Y		
Kv1.4	36	Y		

Table 2: Temperature corrections for models indexed at $0°C$. References provided for models constructed with nonmammalian data.

value was imputed whereever the ambient temperature was not reported directly. This was confirmed via email with a Channelpedia curator.

Temperature data were then corrected through two approaches. When such a model was constructed from a channel expressed in mammals, we assumed $36°C$ as the model's experimental temperature. Examination of one source of nonmammalian data yielded a reference to previous work for baseline experimental conditions, and we were able to find accurate temperatures for the two models in that paper. Corrected values and references are found in Table 2.

2.2 Temperature rescaling

A given conductance model holds exactly at only the temperature for which it was constructed. However, the effect of temperature on rate may be estimated through the ad hoc temperature coefficient Q_{10}, a factor by which reaction rates are scaled when temperature is increased by $10°C$.

$$Q_{10} = \left(\frac{R_2}{R_1}\right)^{(T_2-T_1)/10} \tag{6}$$

Voltage-dependent kinetic rates are described by $1/\tau(V)$ in conductance models, so Q_{10} in terms of τ is:

$$Q_{10} = \left(\frac{\tau_1}{\tau_2}\right)^{(T_2-T_1)/10} \tag{7}$$

and

$$\tau_2 = \tau_1 e^{\frac{logQ}{10}(T_1-T_2)} \tag{8}$$

or

$$\tau_2 = C\tau_1 \tag{9}$$

A slow variable at temperature T_0, then, can become a fast variable at $T_1 > T_0$. However, it is also possible for a channel to have $Q_{10} < 1$, making it faster at low temperatures than at high temperatures, as is the case in some

Experimental Temperature T_1 ($^\circ C$)	$T_1 - T_2$ ($^\circ C$)	$\frac{\tau_2}{\tau_1}$ at $T_2 = 23^\circ C$
9	-14	0.2
20	-3	0.7
21	-2	0.8
22	-1	0.9
23	0	0
24	1	1.1
25	2	1.2
28	5	1.7
34	11	3.3
35	12	3.7
36	13	4.2

Table 3: Model scaling of characteristic time τ for various experimental temperatures (assuming $Q_{10} = 3$).

transient receptor potential (TRP) channels [3]. These authors also cited [8] as noting that Q_{10} tends to take values between 2 and 4 for the voltage-gated channels that are our primary concern here, and we adopt the traditional value of 3 for all channels.

Because Q_{10} imperfectly characterizes rate changes, it would normally be desirable to apply temperature corrections to as few models as possible while remaining in a temperature range suitable for development. We therefore chose the modal temperatures $23^\circ C$ for all of our combined Xenopus models and $36^\circ C$ for the mammalian models. Approximate temperature-induced changes in τ for $Q_{10} = 3$ are in Table 3. Letting $T = 23^\circ C$ will slow variables originally modeled at $36^\circ C$ by approximately fourfold.

It is important to note that half-voltages may also shift with temperature [22], but we do not model this here.

2.3 Selection of models

We removed all ion channel models that did not refer to genetic information (often these models were from older studies). This excluded from consideration all voltage-gated channel models without the x.y descriptor indicating genetic family as well as all M-type potassium channels and L-type calcium channels. We were left with 28 channel models - however, 11 of these were excluded either because they exceeded the scope of our modeling techniques or due to parsing difficulties, leaving 17 models. Further documentation, including remarks on whether the excluded channels are likely to form memories based on our main results, follows:

Index	Type	$T(^{\circ}C)$	τ-scaling	Notes	References
2	Cav2.1	21	0.8	Fast, persistent	[13]
3	Cav2.2	36	4.2		[9]
4	Cav2.3	36	4.2		[13]
5	Cav3.1	25	1.2	Excluded	[20]
6	Cav3.3	25	1.2		[20]
7	HCN1	36	4.2	Persistent	[14]
8	HCN2	36	4.2	Persistent	[14]
9	HCN3	36	4.2	Persistent	[14]
10	HCN4	36	4.2	Persistent	[14]
16	Kir2.1	28	1.7	Fast activation	[11]
17	Kv1.1	24	1.1		[2]
18	Kv1.2	20	0.7		[18]
19	Kv1.3	36	4.2	Excluded	
20	Kv1.4	36	4.2		[19]
21	Kv1.5	9	0.2	Fast activation, Excluded	
22	Kv1.6	23	1		[7]
23	Kv2.1	23	1		[21]
24	Kv2.2	23	1		[16]
25	Kv3.1			Excluded	
26	Kv3.1			Excluded	
27	Kv3.1			Excluded	
28	Kv3.2			Excluded	
29	Kv3.3			Excluded	
30	Kv3.3			Excluded	
31	Kv3.4			Excluded	
32	Kv4.2			Excluded	
43	Nav1.3	23	1	Fast activation	[5]
44	Nav1.6	23	1	Fast, Persistent	[17]

Table 4: Channel models and references, as found in [15]. Indices correspond to those used in the simulations.

2.3.1 Kv3.x

Marom and Abbott [12] argued that Kv3.x channels could not be accurately modeled using only voltage-dependence, and these channels require the addition of a voltage-independent internal state memory. We excluded these models as they are not within the scope of our simulation framework.

2.3.2 Numerical/parsing problems

Kv4.2 was excluded due to parsing difficulties. This channel is an A-type potassium channel that should be expected to have two timescales, one fast and one slow depending on voltage, and it has unknown memory-forming potential. Kv1.5 is fast-activating and may be memory-forming.

Similar parsing difficulties led to exclusion of the Kv1.3 and Ca3.1 channels, although these channels do not have properties that suggest they would form memories (although Ca3.1, like other Ca channels, might preserve Kir memories).

References

[1] Jean Chemin, Arnaud Monteil, Edward Perez-Reyes, Emmanuel Bourinet, Joël Nargeot, and Philippe Lory. Specific contribution of human t-type calcium channel isotypes (α1g, α1h and α1i) to neuronal excitability. *The Journal of physiology*, 540(1):3–14, 2002.

[2] Macdonald J Christie, John P Adelman, James Douglass, and R Alan North. Expression of a cloned rat brain potassium channel in xenopus oocytes. *Science*, 244(4901):221–224, 1989.

[3] Shin-Ho Chung, Olaf S Andersen, and Vikram Krishnamurthy. *Biological membrane ion channels*. Springer, 2007.

[4] Michael Clerx, Paul GA Volders, and Pieter Collins. Myokit: A framework for computational cellular electrophysiology. In *Computing in Cardiology 2014, Cambridge MA USA, 7-10 Sept.*

[5] Theodore R Cummins, Fabio Aglieco, Mathurkrisnan Renganathan, Raimund I Herzog, Sulayman D Dib-Hajj, and Stephen G Waxman. Nav1. 3 sodium channels: rapid repriming and slow closed-state inactivation display quantitative differences after expression in a mammalian cell line and in spinal sensory neurons. *The Journal of Neuroscience*, 21(16):5952–5961, 2001.

[6] Augustus O Grant. Cardiac ion channels. *Circulation: Arrhythmia and Electrophysiology*, 2(2):185–194, 2009.

[7] A Grupe, KH Schröter, JP Ruppersberg, M Stocker, Th Drewes, S Beckh, and O Pongs. Cloning and expression of a human voltage-gated potassium

channel. a novel member of the rck potassium channel family. *The EMBO journal*, 9(6):1749, 1990.

[8] Bertil Hille. *Ionic channels of excitable membranes*, volume 507. Sinauer Sunderland, MA, 2001.

[9] S-J Huang and DW Robinson. Activation and inactivation properties of voltage-gated calcium currents in developing cat retinal ganglion cells. *Neuroscience*, 85(1):239–247, 1998.

[10] Eugene M Izhikevich. *Dynamical systems in neuroscience*. MIT press, 2007.

[11] Samy MY Makary, Tom W Claydon, Decha Enkvetchakul, Colin G Nichols, and Mark R Boyett. A difference in inward rectification and polyamine block and permeation between the kir2. 1 and kir3. 1/kir3. 4 k+ channels. *The Journal of physiology*, 568(3):749–766, 2005.

[12] Shimon Marom and LF Abbott. Modeling state-dependent inactivation of membrane currents. *Biophysical journal*, 67(2):515–520, 1994.

[13] Tsugumichi Miyasho, Hiroshi Takagi, Hideo Suzuki, Shigeo Watanabe, Masashi Inoue, Yoshihisa Kudo, and Hiroyoshi Miyakawa. Low-threshold potassium channels and a low-threshold calcium channel regulate ca 2+ spike firing in the dendrites of cerebellar purkinje neurons: a modeling study. *Brain research*, 891(1):106–115, 2001.

[14] Sven Moosmang, Juliane Stieber, Xiangang Zong, Martin Biel, Franz Hofmann, and Andreas Ludwig. Cellular expression and functional characterization of four hyperpolarization-activated pacemaker channels in cardiac and neuronal tissues. *European Journal of Biochemistry*, 268(6):1646–1652, 2001.

[15] Rajnish Ranjan, Georges Khazen, Luca Gambazzi, Srikanth Ramaswamy, Sean L Hill, Felix Schürmann, and Henry Markram. Channelpedia: an integrative and interactive database for ion channels. *Frontiers in neuroinformatics*, 5, 2011.

[16] Felicitas Schmalz, Jacqueline Kinsella, Sang Don Koh, Fivos Vogalis, Anne Schneider, Elaine RM Flynn, James L Kenyon, and Burton Horowitz. Molecular identification of a component of delayed rectifier current in gastrointestinal smooth muscles. *American Journal of Physiology-Gastrointestinal and Liver Physiology*, 274(5):G901–G911, 1998.

[17] Marianne R Smith, Raymond D Smith, Nicholas W Plummer, Miriam H Meisler, and Alan L Goldin. Functional analysis of the mouse scn8a sodium channel. *The Journal of neuroscience*, 18(16):6093–6102, 1998.

[18] Leslie K Sprunger, Nancy J Stewig, and Scott M O'Grady. Effects of charybdotoxin on k+ channel (kv1. 2) deactivation and inactivation kinetics. *European journal of pharmacology*, 314(3):357–364, 1996.

[19] Walter Stühmer, J Peter Ruppersberg, KH Schröter, Bert Sakmann, Martin Stocker, KP Giese, A Perschke, Ariane Baumann, and Olaf Pongs. Molecular basis of functional diversity of voltage-gated potassium channels in mammalian brain. *The EMBO journal*, 8(11):3235, 1989.

[20] Achraf Traboulsie, Jean Chemin, Marc Chevalier, Jean-François Quignard, Joël Nargeot, and Philippe Lory. Subunit-specific modulation of t-type calcium channels by zinc. *The Journal of physiology*, 578(1):159–171, 2007.

[21] Antonius MJ VanDongen, Georges C Frech, John A Drewe, Rolf H Joho, and Arthur M Brown. Alteration and restoration of k+ channel function by deletions at the n-and c-termini. *Neuron*, 5(4):433–443, 1990.

[22] Fan Yang and Jie Zheng. High temperature sensitivity is intrinsic to voltage-gated potassium channels. *eLife*, 3:e03255, 2014.
